# Fully integrated wearable humidity sensor for respiration monitoring

**DOI:** 10.3389/fbioe.2022.1070855

**Published:** 2022-12-02

**Authors:** Xiaofeng Jin, Lin Zha, Fan Wang, Yongzhong Wang, Xueji Zhang

**Affiliations:** ^1^ School of Life Sciences, Anhui University, Hefei, China; ^2^ Key Laboratory of Human Microenvironment and Precision Medicine of Anhui Higher Education Institutes, Anhui University, Hefei, China; ^3^ Department of Oncology, The Second Hospital of Anhui Medical University, Hefei, China; ^4^ Department of Radiotherapy, The First Affiliated Hospital of Anhui Medical University, Hefei, China; ^5^ School of Biomedical Engineering, Shenzhen University, Shenzhen, China

**Keywords:** wearable biosensor, respiration monitoring, humidity sensor, molybdenum disulfide, electrochemical sensor

## Abstract

Respiration monitoring is a promising alternative to medical diagnosis of several diseases. However, current techniques of respiration monitoring often require expensive and cumbersome devices which greatly limit their medical applications. Here, we present a fully integrated wearable device consisting of a flexible LCP-copper interdigital electrode, a sensing layer and a wireless electrochemical analysis system. The developed humidity sensor exhibits a high sensitivity, a good repeatability and a rapid response/recover time. The long-term stability is over 30 days at different relative humidity. By integrating the flexible humidity sensor with miniaturized electrochemical analysis system (0.8 cm × 1.8 cm), response current concerning respiration can be wirelessly transmitted to App-assisted smartphone in real time. Furthermore, the fabricated humidity sensor can realize skin moisture monitoring in a touch-less way. The large-scale production of miniaturized flexible sensor (4 mm × 6 mm) has significantly contributed to commercial deployment.

## Introduction

Advancements in wearable flexible sensors and smartphone healthcare systems have contributed to the commercial market for wearable technology (Gao et al., 2016; Balakrishnan et al., 2019; Jin X. et al., 2020). At present, fully integrated wearable sensors have been available to consumers as indicators of heartbeats and physical activity, but there are few reports to realize the continuous monitoring of respiration rate with wearable miniaturized sensing platform. Respiration rate, also known as the respiratory frequency, is generally defined as the number of breaths during a minute (Liu et al., 2019). Respiration rate is considered as one of the four primary vital signs of life, along with body temperature, pulse and blood pressure whose abnormity is an essential indicator of serious illness such as cardiac arrest (Maharaj et al., 2015), chronic heart failure (Ponikowski et al., 2001), pneumonia (Rambaud-Althaus et al., 2015), pulmonary embolism (Jimenez et al., 2016), overdose (Hochhausen et al., 2018) and sleep apnea syndrome (SAS) (Liu et al., 2019). Many different techniques have been proposed for respiration monitoring, including spirometry, capnometry, and pneumography. However, current techniques of respiration monitoring usually require complex, costly and cumbersome devices. Therefore, there is a great demand to develop an alternative technology for respiration monitoring. Given the human exhaled air humidity is above 90% relative humidity (RH), an attractive strategy would be to develop a humidity sensor to realize long-term continuous monitoring of respiration. Furthermore, humidity sensor can be easily integrated into wearable devices without any complicated accessory required. For example, Choi et al., presented a wearable humidity sensor using nitrogen-doped graphene fiber for real-time humidity monitoring. The fabricated sensor can detect humidity levels from 2.6% RH to 66.4% RH (Choi et al., 2018). Guder et al. (2016) reported a paper-based humidity sensor attached inside a flexible textile mask for continuous monitoring of the respiratory activity. Such device expanded new possibilities of applications of paper-based sensors as low-cost wearable humidity sensors. One of the drawbacks of this device is that the bending of the paper is prone to causing the sensor to crack. Therefore, there are still several limitations of current wearable humidity sensing technologies for respiration monitoring (Su et al., 2021a; Liu et al., 2022). First, flexible substrate material is a critical factor for wearable applications. It is necessary to use flexible sensors to accommodate the intrinsically flexible of the human body and its inner organisms (Su et al., 2021b; Li et al., 2022). Second, current wearable techniques for respiration monitoring usually require complex, costly and cumbersome devices which greatly limit their medical applications (Su et al., 2022). The proposed humidity sensor provided two promising strategies to meet such a demand. The first strategy was to use robust LCP-copper interdigital electrode (4 mm × 6 mm) which was patterned by flexible printed circuits (FPC) technology that can realize large-scale production as flexible base electrode. Meanwhile, through miniaturized overall system design (0.8 cm × 1.8 cm), the proposed device is easy to wear and cost-effective. We believe our work provides promise in addressing major industrial manufacturing process challenges and can potentially be a good addition to the commercialization of wearable humidity sensor for respiration monitoring.

Recently, transition metal dichalcogenides (TMDs) such as molybdenum disulfide (MoS_2_) (Zhang et al., 2014; Zhang et al., 2016; Ze et al., 2017), rhenium disulfide (ReS_2_) (Adepu et al., 2021), and tungsten disulfide (WS_2_) (Jha and Guha et al., 2016; Chen et al., 2018; Zhang et al., 2018) have gained considerable attention for humidity sensing. As a water-soluble polymer, Polyvinylpyrrolidone (PVP) is not only a promising material for humidity sensing, but also can be used for a suitable stabilizer to ensure the uniform dispersal of MoS_2_ in humidity sensing (Jin X. F. et al., 2020). Otherwise, liquid crystal polymer (LCP) has shown great promise as a soft substrate for wearable humidity sensor due to extremely low moisture absorption, flexibility, chemical inertness, and ease of micro-machining (Ha et al., 2012; Redhwan et al., 2017; Ali Khan et al., 2021). Here we present a fully integrated wearable humidity sensor based on flexible LCP substrate and application for detecting respiration rate and breath patterns. Copper interdigital electrodes were patterned by flexible printed circuits (FPC) technology which is an industrial manufacturing process that can realize large-scale production. LCP was selected as the better choice of flexible substrate for fabricating humidity sensors due to its biocompatibility, chemical inertness, and electromechanical properties (Ha et al., 2012). Using inkjet printing technology, MoS_2_/PVP nanocomposites were deposited on copper interdigital electrode surface as the sensing layers for the detection of humidity gas. Ultimately, we integrated the flexible copper interdigital electrode components, sensing layer, and miniaturized printed circuit board (PCB) board into a wearable sensor for respiration monitoring.

## Materials and methods

### Materials

MoS_2_, absolute ethanol (99.7 wt%) were purchased from Aladdin Ltd. (Shanghai, China). Poly (N-vinylpyrrolidone) (PVP, average Mw 40,000) was purchased from Sigma-Aldrich Co., Ltd. (Shanghai, China). LCP substrate of 50 μm was provided from Taiflex Scientific Co., Ltd. All chemicals were of reagent grade.

### Apparatus

All electrochemical experiments were conducted on a CHI660E electrochemical workstation. Electrode appearance scanning microscopy was handled using VHX-5000 Ultra-depth three-dimensional microscope (keyence, Japan). Scanning electron microscopy (SEM) images were collected on a Sigma HV (Zeiss, Germany). Fourier transform infrared spectroscopy (FT-IR) spectra were collected on a Nicolet iN10 FT-IR spectrometer (Thermo Fisher, United States).

### Preparation of molybdenum disulfide/polyvinylpyrrolidone ink

1.0 g bulk MoS_2_ was suspended in a mixed solution of absolute ethanol and deionized water (1:1, v/v) and subjected to ultrasonication with 380 W power. After exfoliating at room temperature for 1 h, 0.2 g PVP was added as surfactants and stabilizer for another 48 h under the same conditions. Then the suspension was centrifuged for 15 min at 7,500 rpm, and the resulting supernatant was preserved as the MoS_2_/PVP ink for inkjet printing (Jin X. et al., 2020).

### Fabrication of the flexible humidity sensors

The humidity sensor was fabricated on a 50 μm thick LCP substrate with copper cladding on one side. The copper interdigital electrodes were fabricated on the initial flexible substrate by FPC technology, which involves two main steps as followed: First, optical dry film photoresist lithography (DFPL) was performed by roller lamination of the dry film photoresist (30 μm, Dupont) on LCP copper clad plate at 100°C and a speed of 1.4 m/min, followed by UV-exposure with the energy density of 130 mJ/cm^2^. Second, the exposed LCP copper clad plate was etched by an aqueous solution of 0.5 mol/L FeCl_3_ and HCl (1:1).

The as-prepared MoS_2_/PVP ink was inkjet-printed on LCP-copper interdigital electrodes which were pre-treated by oxygen plasma treatment (100 W, 2 min) to fabricate humidity sensors. Inkjet printing was conducted using a commercial drop-on-demand Epson L310 printer. The printer cartridge was designed to dispense droplets with a volume of 3 pL based on the orifice of 63.5 μm in diameter (Jin X. F. et al., 2020). MoS_2_/PVP ink was inkjet-printed on copper interdigital electrodes to fabricate humidity sensors, repeat 10 times and dry at 42°C for 10 min after each printing. The flexible humidity sensors were left to dry for 12 h under ambient conditions.

## Results

### Fabrication and characterization of electrodes

**FIGURE 1:**
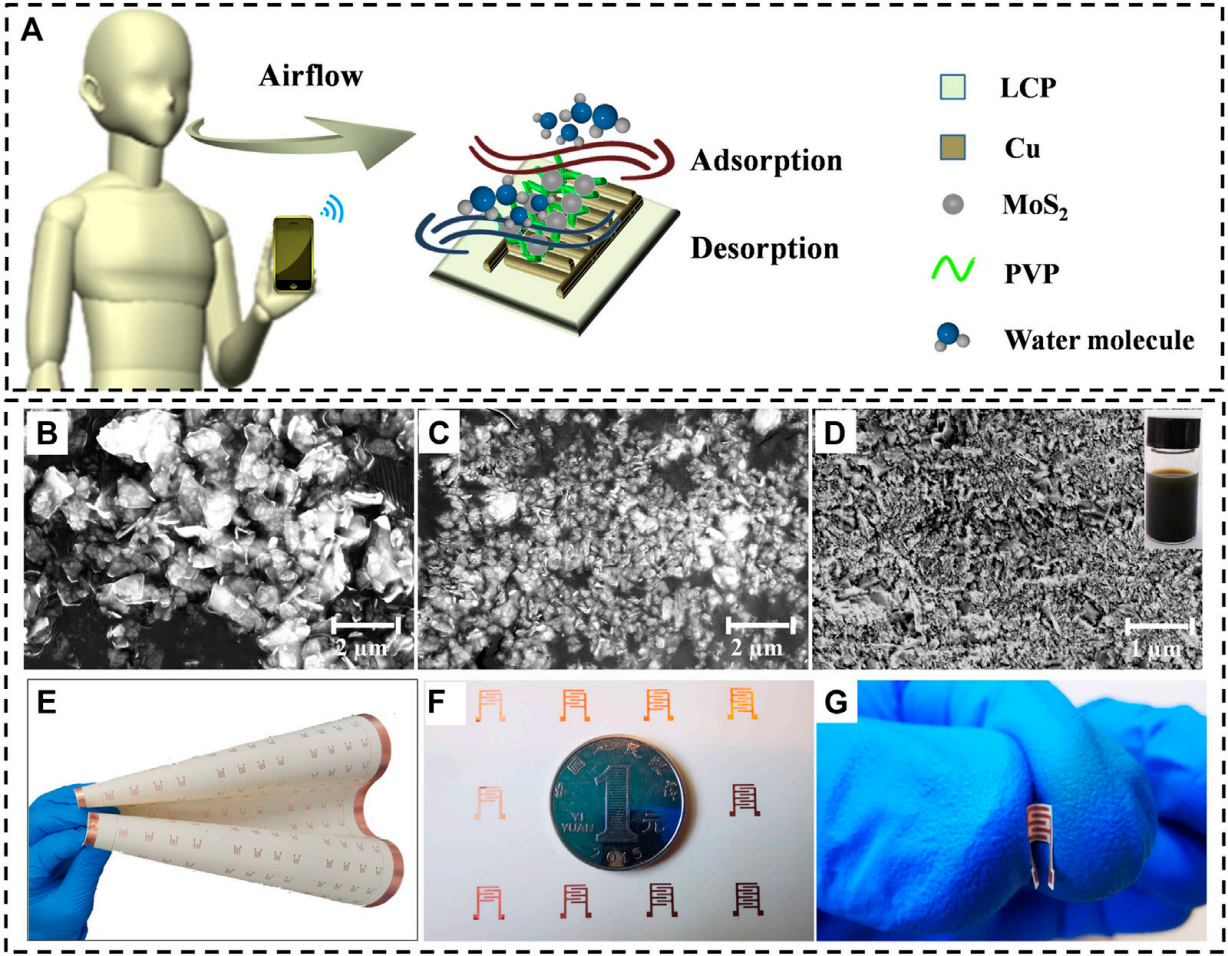
**(A)** Schematic illustration of humidity sensor fabricated and respiratory rate monitoring. SEM images of bulk MoS_2_ without **(B)** and with **(C)** liquid-phase ultrasonic exfoliation, **(D)** PVP-assisted liquid-phase ultrasonic exfoliation of MoS_2_ (the upper right corner is the photograph of MoS_2_/PVP ink), **(E)** Copper interdigital electrode array on flexible LCP substrate, **(F)** Humidity sensor is very small (4 mm × 6 mm), **(G)** Bent interdigitated electrode on flexible LCP substrate.

The humidity sensor fabrication procedure is illustrated in Figure 1A. The interdigital electrodes were fabricated on a mechanically flexible LCP substrate by FPC technology. As initial substrate, a dimension of 250 mm × 300 mm × 50 μm LCP substrate with 18 μm copper cladding on one side was patterned into 180 pieces of miniature interdigital electrode with 4 mm in width and 6 mm in length. As one of two-dimensional (2D) nanomaterials, MoS_2_ possesses an inherently high surface-to-volume ratio which could serve as an ideal candidate for gas sensing (Zhang et al., 2014). Owing to the assisted exfoliation of PVP, MoS_2_/PVP ink (Figure 1D) has the more uniform and smaller sizes than MoS_2_ ink (Figure 1C) which increases the edge active sites. Inset in the top right corner of Figure 1D was the photograph of MoS_2_/PVP ink which is stable for more than 1 month under ambient conditions. As indicated from the SEM image in Figures 1B–D, the size of layered MoS_2_ was considerably reduced to nanometer scale after liquid-phase ultrasonic exfoliation. As shown in Figures 1E–G, the LCP-copper interdigital electrodes exhibited extreme flexibility.

**FIGURE 2:**
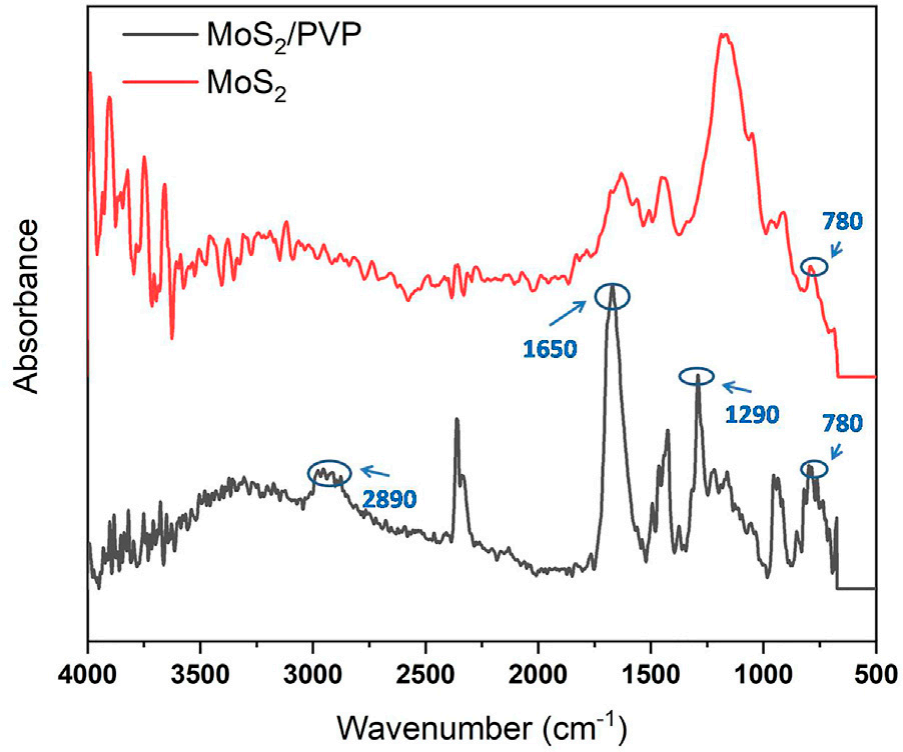
FT-IR spectra of MoS_2_ and MoS_2_/PVP.

Figure 2 gave a comparison of FT-IR spectra between MoS_2_ and MoS_2_/PVP, which further confirmed the successful preparation of MoS_2_/PVP ink. The typical characteristic peaks of PVP were observed in the range of 4,000–500 cm^−1^. The stretching vibration of C-H was seen at 2,890 cm^−1^. The characteristic peak at 1,650 cm^−1^ corresponds to the carbonyl group. The peak at 1,290 cm^−1^ was assigned to the stretching vibration of C-N. The FT-IR spectrum exhibits a characteristic peak at 780 cm^−1^, indicating the existence of a molybdenum compound.

### Analytical performance of humidity sensors

The humidity atmospheres were produced by different saturated salt solutions of LiCl for 11% RH, MgCl_2_ for 33% RH, Mg(NO_3_)_2_ for 54% RH, NaCl for 75% RH, KCl for 84% RH and KNO_3_ for 94% RH, respectively (Dai et al., 2019). The humidity-sensing performances of sensors were evaluated by the response (R) which was defined as: $R=\left(I_{RH}-I_{11}\right)/I_{11}$ , where $I_{RH}$ and $I_{11}$ are the measured current of the sensor at the given RH and 11% RH levels, respectively.

**FIGURE 3:**
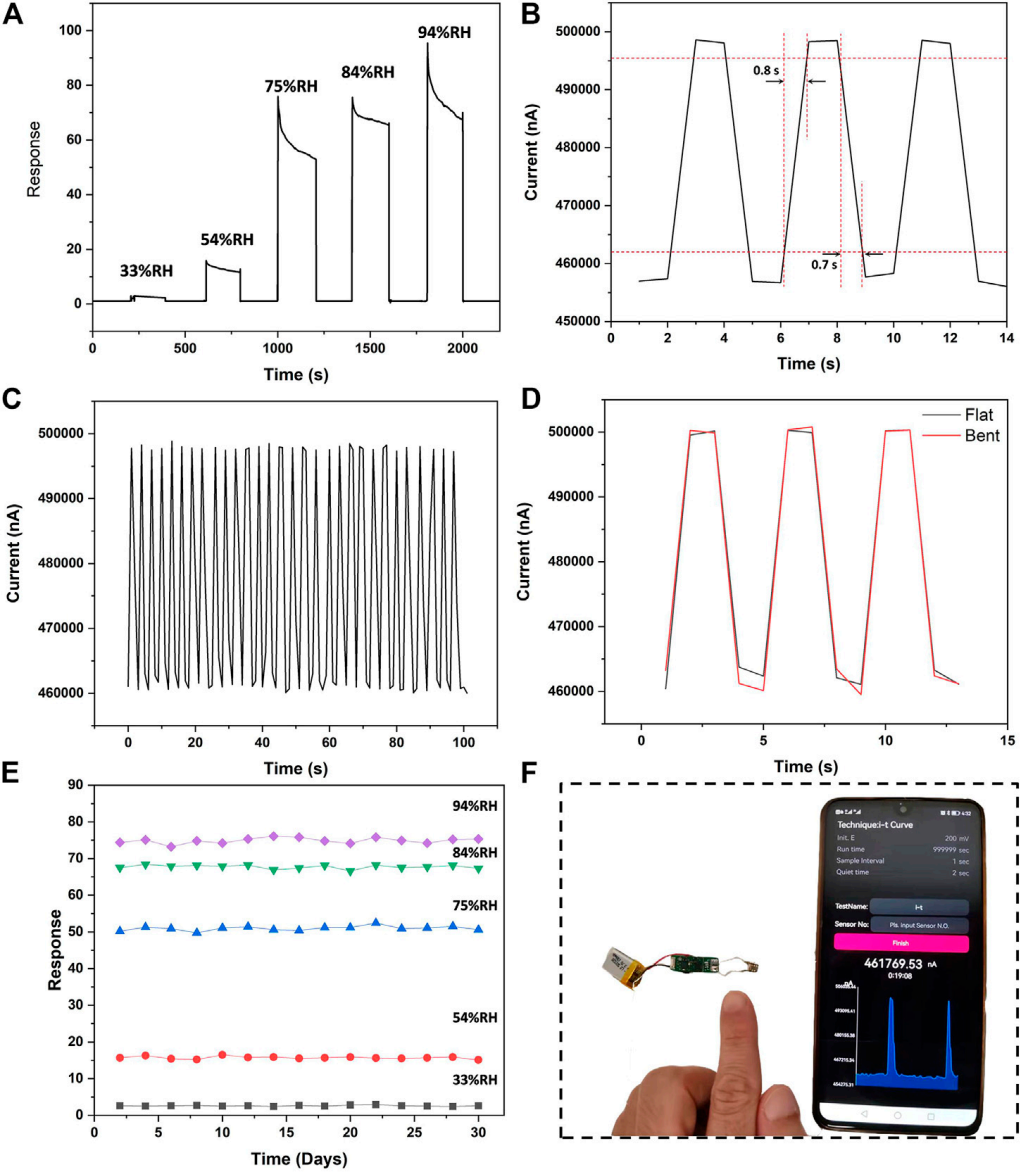
**(A)** Dynamic response of humidity sensors upon exposure to various relative humidity levels, **(B)** Typical breathing pattern for measuring the response/recovery time, **(C)** Continuous response and recovery curve from 55% RH to above 90% RH for 30 cycles, **(D)** Dynamic response of humidity sensors under flat and bent states at bending angle of 60°, **(E)** Responses of humidity sensor every few days towards various relative humidity levels, **(F)** Touch-less skin moisture response from a volutteer’s fingertip.

As shown in Figure 3A, the dynamic response current decreases with humidity increasing when the as-fabricated flexible humidity sensors were exposed to humidity gas from low to high level. The increase in current response could be due to the electrical resistance of the sensors decreasing with the increase of relative humidity. Owing to the great surface-area-to-volume ratio and high carrier mobility, MoS_2_ has been demonstrated to be an effective nanomaterial for humidity sensing. Our previous study has proven the better humidity sensing performance of MoS_2_/PVP sensor compared to the pure MoS_2_ sensor, possibly due to better hydrophilicity of MoS_2_/PVP hybrid nanocomposite, which may contribute to the reduction of mass transfer resistance and consequently promote the reaction kinetic (Jin X. et al., 2020). There are two main kinds of adsorption processes between water molecules and sensing materials, including chemisorption and physisorption. The first adsorption process is chemisorption which is difficult to remove after chemisorbed layer is formed. The second adsorption process is physisorption which will happen above the chemisorbed layer with the increase of the adsorbed water molecules (Ze et al., 2017). The peptide groups of lactams in PVP structure are active binding sites for hydrogen donors. The mechanism of adsorption/desorption of water molecular and PVP is schematically described as follows:

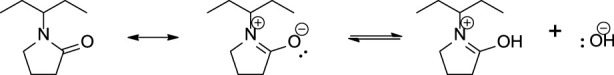

(1)



(1)

At low relative humidity, chemisorption of water molecules occurs on the surface of the sensing layer. These chemisorbed water molecules act as electron donors for the sensing materials, allowing for decreased electrical resistance. However, due to the discontinuous of the water layer at this process, the hydronium ion (H_3_O^+^) transfer is difficult to occur under the electrostatic field. With relative humidity increasing to high level, more water molecules are physiosorbed above the chemisorbed layer by hydrogen bonding to form continuous water multilayer. According to the Grotthuss chain reaction, the protons hopping-transfer is generated and used to ionic conductivity, leading to a rapid decrease in electrical resistance and increase in response current.

Response/recovery time is one of the most crucial parameters to consider for fabricating humidity sensors (Bahuguna et al., 2020). The response and recover times are defined as the time taken from 10% to 90% of the total current change and *vice versa* (Guo et al., 2017). As shown in Figure 3B, it can be measured from the breathing pattern that the as-fabricated humidity sensors have an extremely short response and recovery time of 0.8 and 0.7 s, respectively.

Repeatability and Long-term stability are two core properties to consider for commercial applications of humidity sensors. Considering the application for continuous monitoring of respiration rate, repeatability was evaluated by measuring the time-dependent current response from 55% RH to above 90% RH (breathing pattern). The consistent baseline (current response of device under 45% RH) and the current changes of the device under 30 cycles of exposure to the high relative humidity (above 90% RH) indicate good repeatability of the proposed humidity sensor (Figure 3C). Long-term stability of the humidity sensors was evaluated by measuring the current response towards various relative humidity for every few days. As shown in Figure 3E, the current responses toward various relative humidity remain stable for 30 days, thus to ensure the reliability of respiratory frequency monitoring application. The mechanical robustness of the sensors was evaluated by a bending test which was performed based on the procedure reported by Wu et al. (2019). Figure 3D shows that no noticeable response degradation was observed at bending angle of 60°, indicating that bending tests do not adversely affect the performance of the proposed humidity sensors.

The normal respiration of an adult lasts 3–5 s and exhaled air humidity is above 90% RH. In consideration of the high performance of the humidity sensors fabricated in this study, we further integrated the flexible copper interdigital electrode components, sensing layer, and miniaturized PCB board into a wearable patch sensor for respiration monitoring.

The PCB board of electrochemical workstation with miniaturized format (0.8 cm × 1.8 cm) consists of a signal conditioning part, a Bluetooth transceiver and a programmable electrochemical chip. The signal is wireless communication between wearable humidity sensor and smartphone-based platforms. The system is powered by a 150 mAh rechargeable lithium battery. The applied potential was set as 200 mV during operation. Due to a low soldering temperature of LCP (260°C–310°C, 10 s), conductive silver paste was used for the connection between wire and LCP-copper interdigital electrode.

**FIGURE 4:**
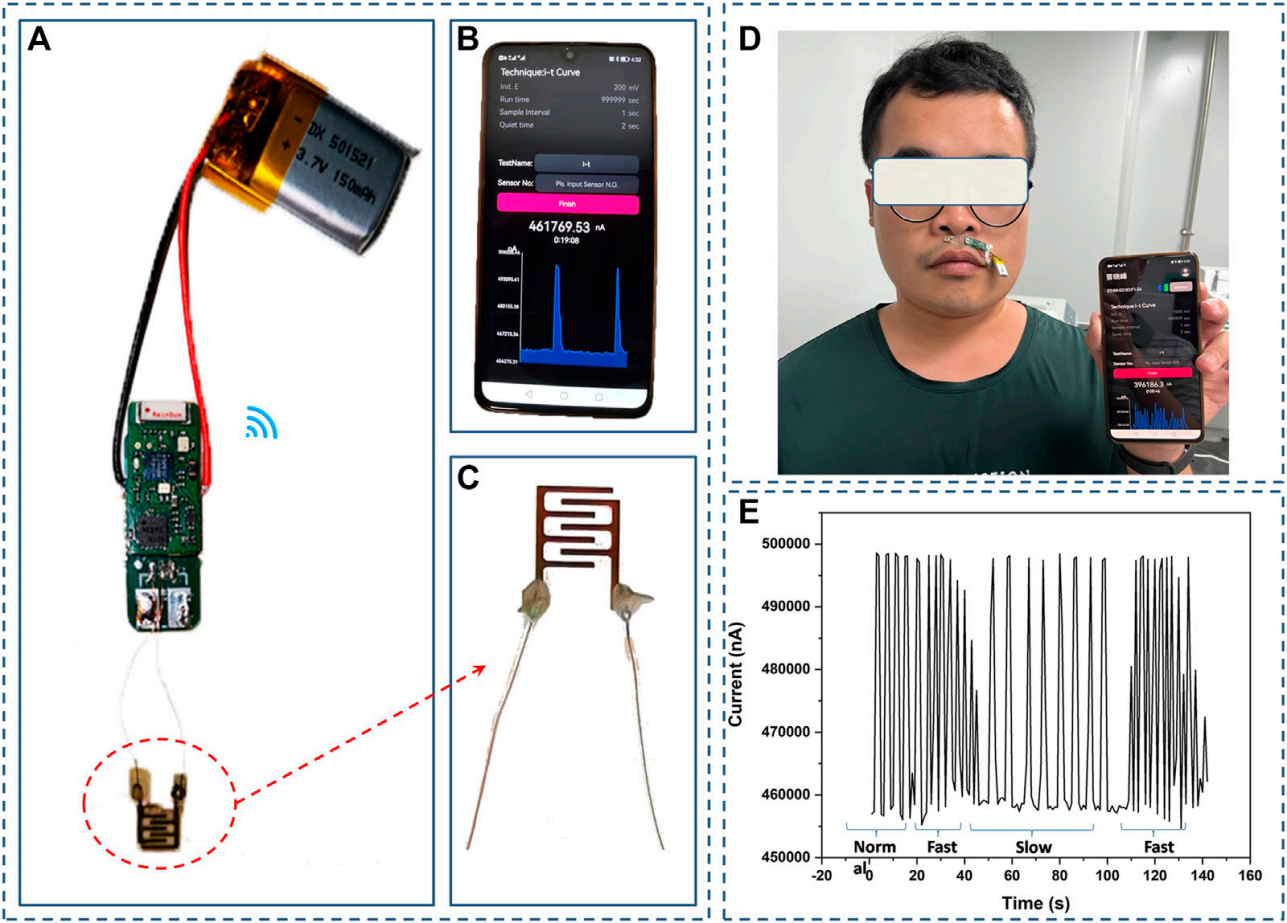
**(A)** Photograph of the fully integrated wearable humidity sensor, **(B)** Photograph of App-assisted smartphone as a wireless receptor, **(C)** Photograph of humidity sensor connected by conductive silver paste between wire and LCP-copper interdigital electrode, **(D)** Photograph of the device attached under a volutteer’s nose, **(E)** The device can distinguish different respiration patterns.

In order to evaluate the real-time breath monitoring application of the fabricated humidity sensor, the device was attached under a volunteer’s nose about 5 mm. As shown in Figure 4, the device can distinguish different respiration patterns such as normal breathing, fast breathing and slow breathing with good stability and sensitivity, holding great promise for realizing continuous monitoring of breath. In addition, the fabricated humidity sensor can realize skin moisture monitoring in a touch-less way. At a distance of 2 mm, the sensor showed high sensitivity towards the moisture content of a volunteer’s fingertip, exhibiting its potential applications in non-contact switch, skin examination and spatial localization monitoring (Figure 3F).

## Discussion

In this study, we have developed a fully integrated flexible humidity sensor that enables wearable respiration monitoring. LCP proved to be one of the most desirable flexible materials for humidity sensing applications as its excellent chemical properties, electrical insulator and ultra-low water absorption. The humidity sensing material was prepared by simple liquid-phase ultrasonic exfoliation method. PVP-assisted exfoliation has considerably reduced the dimension of MoS_2_ to nanoscale, increased the surface volume ratio and the edge active sites. Meanwhile, the lactam units of PVP are active binding sites for water molecules which enhance the mobility of charge carriers during polarization. Compared with other humidity sensors for respiration monitoring in literatures (Table 1), the developed humidity sensor exhibited a high sensitivity, a good repeatability and a rapid response/recover time. The responses current were stable over a 30-day test period at different relative humidity. Furthermore, the flexible humidity sensor has been successfully integrated on a wearable device for continuous respiratory monitoring. Due to the industrial manufacturing process of LCP-copper interdigital electrodes, the humidity sensor is sufficiently low-cost that it can be considered for at-home monitoring.

**TABLE 1:** Sensing performances of humidity sensors for respiration monitoring in literatures.

Active materials	Detection range (%RH)	Response time (s)	Recovery time (s)	References
Pt-nRGO fiber	2.6–66.4	2	2	Choi et al. (2018)
ReS_2_ nanosheets	43–95	142.94	35.45	Adepu et al. (2021)
R-GO/PU	10–70	3.5	7	Trung et al. (2017)
WS_2_	up to 90	5	6	Guo et al. (2017)
Ni-Co-P hollow nanobricks	0–97.5	95	27	Chen et al. (2022)
SnSe_2_/MWCNT nanohybrids	10–70	1.8	2.9	Tannarana et al. (2020)
MoS_2_/SnO_2_	0–97	5	13	Zhang et al. (2016)
MoS_2_/PVP	11–94	0.8	0.7	This work

## Data Availability

The original contributions presented in the study are included in the article/Supplementary Material, further inquiries can be directed to the corresponding authors.
